# Effect of Problem-solving Treatment on Self-reported Disability Among Veterans With Gulf War Illness: A Randomized Clinical Trial

**DOI:** 10.1001/jamanetworkopen.2022.45272

**Published:** 2022-12-06

**Authors:** Lisa M. McAndrew, Karen S. Quigley, Shou-En Lu, David Litke, Joseph F. Rath, Gudrun Lange, Susan L. Santos, Nicole Anastasides, Beth Ann Petrakis, Lauren Greenberg, Drew A. Helmer, Wilfred R. Pigeon

**Affiliations:** 1 War Related Illness and Injury Study Center, Veterans Affairs (VA) New Jersey Health Care System, East Orange; 2 Bedford VA Medical Center, Bedford, Massachusetts; 3 Department of Psychology, Northeastern University, Boston, Massachusetts; 4 Department of Biostatistics and Epidemiology, Rutgers School of Public Health, Piscataway, New Jersey; 5 Department of Rehabilitation Medicine, New York University School of Medicine, New York; 6 VA Palo Alto Health Care System, Palo Alto, California; 7 Center for Innovations in Quality, Effectiveness and Safety at Michael E. DeBakey VA Medical Center and Baylor College of Medicine, Houston, Texas; 8 Center of Excellence for Suicide Prevention, Canandaigua VA Medical Center, Canandaigua, New York; 9 Department of Psychiatry, University of Rochester Medical Center, Rochester, New York

## Abstract

**Importance:**

Few evidence-based treatments are available for Gulf War illness (GWI). Behavioral treatments that target factors known to maintain the disability from GWI, such as problem-solving impairment, may be beneficial. Problem-solving treatment (PST) targets problem-solving impairment and is an evidence-based treatment for other conditions.

**Objective:**

To examine the efficacy of PST to reduce disability, problem-solving impairment, and physical symptoms in GWI.

**Design, Setting, and Participants:**

This multicenter randomized clinical trial conducted in the US Department of Veterans Affairs compared PST with health education in a volunteer sample of 511 Gulf War veterans with GWI and disability (January 1, 2015, to September 1, 2019); outcomes were assessed at 12 weeks and 6 months. Statistical analysis was conducted between January 1, 2019, and December 31, 2020.

**Interventions:**

Problem-solving treatment taught skills to improve problem-solving. Health education provided didactic health information. Both were delivered by telephone weekly for 12 weeks.

**Main Outcomes and Measures:**

The primary outcome was reduction from baseline to 12 weeks in self-report of disability (World Health Organization Disability Assessment Schedule). Secondary outcomes were reductions in self-report of problem-solving impairment and objective problem-solving. Exploratory outcomes were reductions in pain, pain disability, and fatigue.

**Results:**

A total of 268 veterans (mean [SD] age, 52.9 [7.3] years; 88.4% male; 66.8% White) were randomized to PST (n = 135) or health education (n = 133). Most participants completed all 12 sessions of PST (114 of 135 [84.4%]) and health education (120 of 133 [90.2%]). No difference was found between groups in reductions in disability at the end of treatment. Results suggested that PST reduced problem-solving impairment (moderate effect, 0.42; *P* = .01) and disability at 6 months (moderate effect, 0.39; *P* = .06) compared with health education.

**Conclusions and Relevance:**

In this randomized clinical trial of the efficacy of PST for GWI, no difference was found between groups in reduction in disability at 12 weeks. Problem-solving treatment had high adherence and reduced problem-solving impairment and potentially reduced disability at 6 months compared with health education. These findings should be confirmed in future studies.

**Trial Registration:**

ClinicalTrials.gov Identifier: NCT02161133

## Key Points

**Question:**

What is the efficacy of problem-solving treatment (PST) for veterans with Gulf War illness?

**Findings:**

This randomized clinical trial of 268 veterans found no differences between PST and health education in reduction in disability at the end of the intervention (primary outcome). Results suggested that PST reduced problem-solving impairment (moderate effect, 0.42) and disability at follow-up (moderate effect, 0.39) compared with control.

**Meaning:**

In this trial, PST did not improve the primary outcome compared with health education but improved some outcomes and was acceptable to veterans with Gulf War illness.

## Teaser

This randomized clinical trial of veterans with Gulf War illness examines whether problem-solving treatment is efficacious in reducing disability and problem-solving impairment.

## Introduction

Persistent, medically unexplained physical symptoms disproportionately burden individuals exposed to war.^1,2^ As many as 30% of military veterans of the Persian Gulf War (1990-1991) developed chronic disabling symptoms, collectively referred to as chronic multisymptom illness or Gulf War illness (GWI).^1,2,3^

The 2021 Clinical Practice Guidelines for the Management of Chronic Multisymptom Illness recommend cognitive behavioral treatment^4,5,6^ based on a clinical trial among veterans with GWI and indirect evidence from multiple trials for related conditions.^7^ More direct evidence is needed to support the efficacy of behavioral treatments that target mechanisms relevant for GWI and are acceptable to veterans.^4,8,9^ Developing acceptable treatments for GWI is critical because nonspecific treatments may not be acceptable to veterans who fought to legitimize GWI.^10,11^

One factor known to maintain the disability of GWI is impairment in problem-solving ability, an executive function defined as the ability to find solutions to problems without an easily identified solution.^12,13^ Impairment in problem-solving increases disability because it makes it difficult to overcome problems that affect daily activities^14^ and effectively manage chronic conditions, such as GWI.^15^ Problem-solving treatment (PST) is a cognitive behavioral treatment that remediates problem-solving impairment for other conditions (eg, traumatic brain injury).^16,17^ We performed a randomized clinical trial to examine the efficacy of telephone-delivered PST compared with an active control, telephone-delivered health education (HE), for reducing the disability and problem-solving impairment of veterans with GWI.

## Methods

### Procedure

This randomized clinical trial, conducted between January 1, 2015, and September 1, 2019, was a parallel-group, individually randomized trial with 1:1 allocation that compared telephone-delivered PST with telephone-delivered HE.^18^ Veterans with GWI were recruited nationwide with emphasis on local recruitment at the 3 study sites, each with local institutional review board approval. Veterans were screened via telephone to determine eligibility. Eligible veterans provided written informed consent. Near the end of the study, veterans who were unable to travel to 1 of the 3 sites could be mailed the written consent form. Research personnel at the primary site conducted all treatment sessions. Treatment was telephone delivered because disability from GWI can make in-person appointments difficult, and substantial research supports the efficacy of telephone-delivered behavioral treatments.^19,20,21^ The trial ended when sample size was reached. This clinical trial follows the Consolidated Standards of Reporting Trials (CONSORT) reporting guideline, and no changes to the outcomes, assessments, inclusion criteria, or treatments were made after the start of data collection.^22^ The full trial protocol can be found in Supplement 1.

### Participants

Participants were included if they were deployed to the Persian Gulf War (August 1990 to November 1991), met the Kansas definition for GWI,^2^ and scored at least half an SD worse than the mean on the World Health Organization Disability Assessment Schedule (WHODAS 2.0).^23^ Participants were excluded if they had current suicidal or homicidal intent or plan, schizophrenia or current psychotic symptoms, a disability that would preclude telephone treatment, or self-reported diagnosis of a degenerative brain disorder or serious psychiatric or medical illness that could limit generalizability of the findings, limit safety, or account for the symptoms of GWI.

### Problem-solving Treatment

Telephone-delivered PST included 12 one-hour sessions using a workbook and was modeled after established PSTs and tailored for veterans with GWI.^16,24^ Veterans were taught how to develop a positive mindset around problem-solving (“I can solve problems”). Veterans were also taught a 5-step approach to problem-solving. Veterans were supported to increase participation in activities of their choosing. Materials for both treatments are available from the corresponding author.

### Health Education

The active control, telephone-delivered HE, included 12 sessions lasting up to 1 hour (typically approximately 40 minutes) using a workbook and was modeled after HE provided in a US Department of Veterans Affairs (VA) specialty clinic.^25^ Sessions were highly structured and emphasized the learning of key health concepts. Study practitioners did not provide behavioral change support.

### Study Practitioners

Study practitioners delivered both interventions and were licensed mental health practitioners or postdoctoral trainees. These practitioners trained for at least 3 days and received peer group (2 times per week) and individual (once per week) supervision. Supervision focused on practitioner competency, adherence to both treatments, and treatment differentiation and included listening to taped sessions, reviewing treatment manuals, and discussing cases. Training included listening to taped sessions, reviewing treatment manuals, and discussing cases.

### Treatment Fidelity

The PST and HE sessions were audiorecorded. We developed fidelity instruments to code sessions for fidelity to session-specific content (range, 0-100%). Selected HE sessions were coded with the PST fidelity instrument to ensure sessions did not include elements of PST. Multiple coders discussed coding inconsistencies until they reached agreement.

### Randomization

Participants were randomized to PST or HE (1:1 ratio) using an urn randomization procedure in which matching was based on disability level and sex at each study site to ensure equitable distribution between groups.^26^ The statistician (S.-E.L.) generated the randomization sequence, and the study coordinator assigned participants to interventions.

### Assessment Methods

Throughout the study, assessments could be completed in person, by mail, or over the telephone, and participants were compensated for completing the assessments. Veterans who were mailed the written consent form did not complete the neuropsychological assessment, because it had to be completed in person (n = 26). Veterans were assessed at baseline, 4 weeks, 12 weeks, and 6 months. Assessors and investigators were blinded to randomization.

### Outcome Measures

The primary outcome was reduction in disability score (WHODAS 2.0) between baseline and 12 weeks; a secondary outcome was reduction in WHODAS 2.0 score between baseline and 6 months. The WHODAS 2.0 measures disability attributable to health conditions^23^ and reflects 2 underlying constructs: activity limitations and participation deficits. Higher scores indicate more disability (range, 1-100). The 12-item measure was used at screening^23^ and the 36-item measure at 4 weeks, 12 weeks, and 6 months. Additional secondary outcome measures were reductions in self-reported problem-solving impairment between baseline and 12 weeks and between baseline and 6 months with the Problem Solving Inventory,^27^ where higher scores indicate greater problem-solving impairment (range, 32-192). Reduction in objective problem-solving impairment between baseline and 12 weeks was assessed with a composite score (mean *z* scores) of performance on a Stroop Color and Word Test (standardized interference score),^28^ Trail Making Test B standardized score,^29^ Halstead Category Test–Russell revised,^30^ and Conners Continuous Performance Test 3 days’ standardized score.^31^ Lower scores indicate greater problem-solving impairment.

Our a priori exploratory outcomes were reduction between baseline and 12 weeks in the Multidimensional Pain Inventory 3-item pain scale (higher scores indicate greater pain; range, 0-18), the Pain Disability Index (higher scores indicate greater disability; range, 0-70),^32^ and the Fatigue Severity Scale (higher scores indicate greater fatigue severity; range, 9-63).^33^ The 6-month pain and fatigue outcomes were not registered but are provided here for context. Participants also completed a short assessment of treatment satisfaction.^34^

### Participant Characterization

The Kansas definition of GWI requires that veterans endorse moderately severe and/or multiple symptoms that started during or after the Gulf War in at least 3 of 6 domains: fatigue; pain; neurologic, cognitive, or mood; skin; gastrointestinal; and respiratory.^2^ Patients with chronic conditions (eg, cancer) that can have diverse symptoms or interfere with respondents’ ability to accurately report their symptoms (eg, psychosis) are excluded.^2^ To improve generalizability in this aging population, we only excluded participants with a disorder that could clearly account for the symptoms of GWI (eg, multiple sclerosis). Participants also completed the Posttraumatic Checklist,^35^ the Patient-Health Questionnaire depression subscale,^36^ and demographic questions, with race and ethnicity classified by the veteran using predefined options (American Indian, Asian, Black, Latinx, Native Hawaiian, White, >1 race or ethnicity, or unknown) to characterize the sample.

### Sample Size

We powered the study to test an effect size Cohen *d* of 0.38 based on a prior clinical trial of older patients with depression^37^ and the assumption that the intraparticipant correlation between the baseline and end of treatment assessment would be approximately 0.5. These assumptions led to a sample size estimate of 109 participants per group to test an effect size Cohen *d* = 0.38 with 80% power and α = .05 (2-sided). After accounting for approximately 15% attrition, we planned to recruit 129 participants per group, 258 in total.

### Statistical Analysis

The primary statistical analysis was conducted between January 1, 2019, and December 31, 2020, with additional sensitivity analysis conducted in 2022. Analyses were performed on an intention-to-treat basis following our protocol and trial registry. Statistical significance was set at a 2-sided *P* < .05. We calculated means (SDs) and compared baseline demographic variables, depression, and posttraumatic stress symptoms between groups to determine the need for any covariates in the analysis for preexisting group differences. No differences required control.

We analyzed the data using a repeated mixed-model analysis, with participants nested within therapist, which was modeled as a random effect. In the first model, the WHODAS 2.0 summary score was treated as the dependent variable, and treatment assignment (PST vs HE), time (baseline, 4 weeks, 12 weeks, and 6-month follow-up) and treatment × time interactions were modeled as fixed effects. Linear contrasts were constructed to evaluate the reduction in disability for each treatment and between treatments at 12 weeks (primary end point) and 6 months (secondary end point). The same mixed-model analysis strategy was applied to address our secondary outcome of problem-solving impairment (self-reported and objective) and our planned exploratory analyses. We report the effect size (Cohen *d*) for each outcome.

Mixed-model analysis was used to assess whether PST produced greater reduction in disability through its effect on reducing problem-solving impairment. The indirect effect of self-reported problem-solving impairment was tested using the CI approach.^38^ A 97.5% CI was constructed for each indirect effect at 12 weeks and 6 months, after Bonferroni adjustment. If 0 was not included in the 97.5% CI, we considered the mediational relationship to be established. We also calculated the proportion of the total effect that was accounted for by the indirect effect (proportion mediated [P_M_]) at 12 weeks and 6 months.

To address missing data, we conducted sensitivity analyses using baseline and multiple imputations.^39^ Baseline imputation assumes that individuals with missing outcome variables at follow-up returned to baseline values. Thus, baseline imputation imputes the missing values of each outcome with the patient’s baseline values. For multiple imputation, we assumed missing at random^39^ and used the Markov chain Monte Carlo approach to impute missing data. Ten imputed data sets were generated using PROC MI in SAS software, version 9.4 (SAS Institute Inc).^40^ Analyses were performed on each imputed data set, with combined estimates calculated using the Rubin rule. PROC Mixed in SAS software, version 9.4 (SAS Institute Inc) was used to perform the mixed-model analysis.

## Results

We screened 511 veterans, of whom 268 were randomized to PST (n = 135) or health education (n = 133) treatment (Figure). Participants’ mean (SD) age was 52.9 (7.3) years; 237 were male (88.4%) and 31 female (11.6%); 12 were American Indian (4.5%), 3 were Asian (1.1%), 63 were Black (23.5%), 18 were Latinx (6.6%), 1 was Native Hawaiian (0.4%), 179 were White (66.8%), 8 were of more than 1 race or ethnicity (3.0%), and 2 were of unknown race or ethnicity (0.7%). Our sample was generally demographically representative of the population of Gulf War veterans (Table 1 and Table 2).

**Figure.**
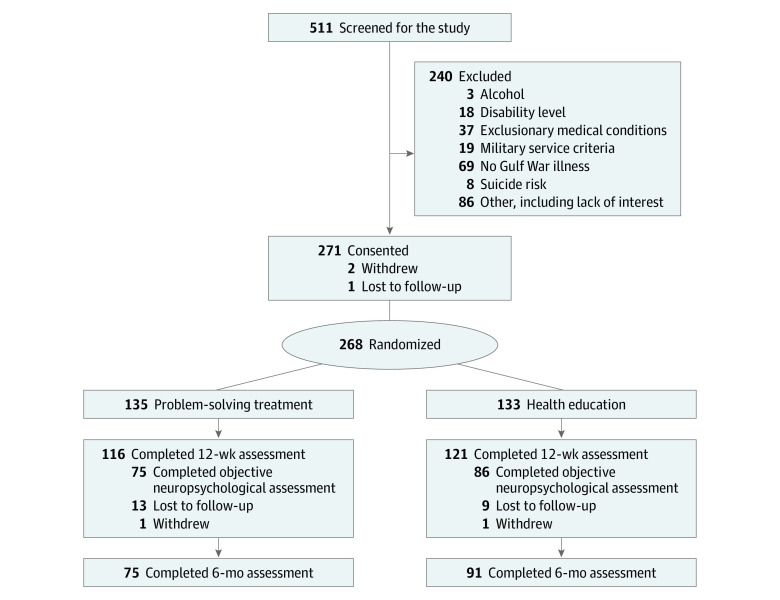
Study Flow Diagram

**Table 1.** Characterization of Participants at Baseline^a^

Characteristic	Total sample (N = 268)	Problem-solving treatment group (n = 135)	Health education group (n = 133)
Age, mean (SD), y	52.9 (7.3)	53.1 (7.6)	52.8 (7.0)
Sex			
Female	31 (11.6)	15 (11.1)	16 (12.0)
Male	237 (88.4)	120 (88.9)	117 (88.0)
Race and ethnicity			
American Indian	12 (4.5)	6 (4.4)	6 (4.5)
Asian	3 (1.1)	2 (1.5)	1 (0.8)
Black	63 (23.5)	30 (22.2)	33 (24.8)
Latinx	18 (6.6)	9 (6.7)	9 (6.8)
Native Hawaiian	1 (0.4)	1 (0.7)	0
White	179 (66.8)	91 (67.4)	88 (66.2)
>1 Race or ethnicity	8 (3.0)	5 (3.7)	3 (2.3)
Unknown^b^	2 (0.7)	0	2 (1.5)
Posttraumatic Checklist score, mean (SD)	36.6 (19.6)	37.0 (20.0)	36.2 (19.2)
Patient-Health Questionnaire depression subscale score, mean (SD)	11.9 (5.7)	11.2 (5.6)	12.4 (5.8)

^a^ Data are presented as number (percentage) of patients unless otherwise indicated.

^b^ Veteran self-report of unknown.

**Table 2.** Effects Within the Treatment Groups

Measure	Mean (SE) scores												
	Problem-solving treatment						Health education						
	Baseline	4 wk	12 wk	6 mo	Change, baseline to 12 wk	Change, baseline to 6 mo	Baseline	4 wk	12 wk	6 mo	Change, baseline to 12 wk	Change, baseline to 6 mo
Disability^a^	46.7 (1.9)	42.5 (2.0)	43.9 (2.0)	44.1 (2.2)	−2.8 (1.2)	−2.6 (1.5)	45.1 (1.9)	44.6 (2.0)	42.8 (2.0)	46.2 (2.1)	−2.2 (1.1)	1.1 (1.3)
Self-reported problem-solving impairment^b^	96.8 (2.5)	94.3 (2.6)	84.1 (2.6)	89.7 (2.9)	−12.7 (1.8)	−7.0 (2.1)	98.0 (2.5)	95.5 (2.6)	91.5 (2.6)	98.3 (2.7)	−6.5 (1.7)	0.3 (1.9)
Objective problem-solving^c^	47.8 (0.4)	NA	48.4 (0.5)	NA	0.8 (0.5)	NA	47.5 (0.4)	NA	48.8 (0.5)	NA	1.4 (0.4)	NA
Pain^d^	3.7 (0.1)	NA	3.7 (0.1)	3.7 (0.2)	0.0 (0.1)	0.0 (0.1)	3.6 (0.1)	NA	3.5 (0.1)	3.6 (0.2)	−0.1 (0.1)	0.1 (0.1)
Pain disability^e^	35.2 (1.5)	NA	33.2 (1.6)	37.1 (1.8)	−2.1 (1.1)	1.8 (1.4)	35.1 (1.5)	NA	34.4 (1.6)	38.3 (1.7)	−0.7 (1.1)	3.2 (1.3)
Fatigue^f^	48.3 (1.3)	NA	45.5 (1.3)	46.2 (1.5)	−2.7 (1.0)	−2.1 (1.2)	46.5 (1.3)	NA	45.7 (1.3)	45.9 (1.4)	−0.8 (1.0)	−0.6 (1.1)

Abbreviation: NA, not applicable.

^a^ Self-reported disability was assessed with the World Health Organization Disability Assessment Schedule 2.0. Higher scores indicate more disability (range, 1-100). Primary outcome was disability at 12 weeks.

^b^ Self-reported problem-solving impairment was assessed with the Problem Solving Inventory. Higher scores indicate greater problem-solving impairment (range, 32-192).

^c^ Objective problem-solving impairment was assessed with a composite score (mean *z* scores) of performance on a Stroop Color and Word Test, Trail Making Test B, Halstead Category Test–Russell revised, and Conners Continuous Performance Test. Lower scores indicate greater problem-solving impairment.

^d^ Pain was assessed with the Multidimensional Pain Inventory 3-item pain scale. Higher scores indicate greater pain (range, 0-18).

^e^ Pain disability was assessed with Pain Disability Index. Higher scores indicate greater disability (range, 0-70).

^f^ Fatigue was assessed with the Fatigue Severity Scale. Higher scores indicate greater fatigue severity (range, 9-63).

Ten percent of veterans (n = 28) were randomly selected to have all their sessions coded for fidelity. There were 336 sessions and 4 were inaudible, resulting in 332 sessions being rated. The average fidelity to PST session-specific content (n = 167 sessions rated) was 97%. The average fidelity to HE session-specific content was 98%, and 99% of HE sessions were 100% differentiated from PST.

There was high adherence and satisfaction with the treatments. Adherence was similar between treatments (χ^2^ = 2.0; *P* = .16); 114 veterans (84.4%) randomized to PST attended all 12 sessions (mean [SD] number of sessions completed, 10.7 [3.3]), and 120 veterans (90.2%) randomized to HE attended all 12 sessions (mean [SD] number of sessions completed, 10.7 [3.3]). Satisfaction was similar for PST (mean [SD], 28.3 [1.8]) and HE (mean [SD], 28.0 [1.8]), with a mean (SD) difference between groups of 0.3 (0.3) (*t* = −1.4; *P* = .17). Veterans in this study had complex health concerns; 22 adverse events occurred in the PST group and 30 in the HE group. Three of the adverse events (increase in psychological symptoms in all 3) were considered potentially attributable to the study (1 in the PST group and 2 in the HE group).

### Disability

The overall treatment × time interaction for disability (WHODAS 2.0) across time points (*F*_3,569_ = 2.62; *P* = .050) suggested that the changes in disability scores over time differed between PST and HE. The primary outcome was change in disability from baseline to 12 weeks. Both PST and HE had small reductions in disability at 12 weeks (PST: baseline mean [SE], 46.7 [1.9]; 12-week mean [SE], 43.9 [2.0]; Cohen *d* = 0.2, *P* = .02; HE: baseline mean [SE], 45.1 [1.9]; 12-week mean [SE], 42.8 [2.0]; Cohen *d* = 0.2, *P* = .051) (Table 2). No difference was found in disability reduction between treatments at 12 weeks (Cohen *d* = 0.1, *P* = .71) (Table 3), which suggested that PST did not reduce disability to a greater degree than HE at 12 weeks. This result was supported by sensitivity analyses (Table 4).

**Table 3.** Between-Treatment Group Effects: Intracorrelations for Therapist and Participant

Measure	Change in PST vs change in HE at 12 wk, mean (SE)	Effect size (Cohen *d*)	Change in PST vs change in HE at 6 mo, mean (SE)	Effect size (Cohen *d*)	Intracorrelation^a^	
					Therapist	Participant
Disability^b^	−0.6 (1.6)	0.06	−3.7 (2.0)	0.4	0.01	0.79
Self-reported problem-solving impairment^c^	−6.2 (2.5)	0.3	−7.3 (2.9)	0.4	0.00	0.79
Objective problem-solving^d^	−0.6 (0.6)	0.1	NA	NA	0.00	0.64
Pain^e^	0.1 (0.1)	0.03	−0.0 (0.2)	0	0.00	0.77
Pain disability^f^	−1.4 (1.6)	0.1	−1.4 (1.9)	0.2	0.00	0.75
Fatigue^g^	−1.9 (1.4)	0.2	−1.5 (1.6)	0.2	0.00	0.77

Abbreviations: HE, health education; NA, not applicable; PST, problem-solving treatment.

^a^ Intratherapist and intraparticipant correlations were estimated using mixed-model analysis.

^b^ Self-reported disability was assessed with the World Health Organization Disability Assessment Schedule 2.0. Higher scores indicate more disability (range, 1-100). Primary outcome was disability at 12 weeks.

^c^ Self-reported problem-solving impairment was assessed with the Problem Solving Inventory. Higher scores indicate greater problem-solving impairment (range, 32-192).

^d^ Objective problem-solving impairment was assessed with a composite score (mean *z* scores) of performance on a Stroop Color and Word Test, Trail Making Test B, Halstead Category Test–Russell revised, and Conners Continuous Performance Test. Lower scores indicate greater problem-solving impairment.

^e^ Pain was assessed with the Multidimensional Pain Inventory 3-item pain scale. Higher scores indicate greater pain (range, 0-18).

^f^ Pain disability was assessed with the Pain Disability Index. Higher scores indicate greater disability (range, 0-70).

^g^ Fatigue was assessed with the Fatigue Severity Scale. Higher scores indicate greater fatigue severity (range, 9-63).

**Table 4.** Imputation Analyses

Variable	Imputation, mean (SE)							
	Multiple				Baseline			
	Change in PST vs change in HE at 12 wk	*P* value	Change in PST vs change in HE at 6 mo	*P* value	Change in PST vs change in HE at 12 wk	*P* value	Change in PST vs change in HE at 6 mo	*P* value
Disability^a^	−0.1 (1.7)	.94	−2.9 (1.8)	.11	−0.4 (1.4)	.78	−2.4 (1.1)	.04
Self-reported problem-solving impairment^b^	−6.0 (2.5)	.02	−6.0 (3.0)	.045	−5.3 (2.3)	.02	−3.7 (1.7)	.03
Objective problem-solving^c^	−0.2 (0.6)	.72	NA	NA	−0.3 (0.5)	.36	NA	NA
Pain^d^	0.03 (0.1)	.81	−0.04 (0.1)	.82	0.03 (0.1)	.79	−0.02 (0.1)	.88
Pain disability^e^	−1.06 (1.6)	.54	−0.7 (2.1)	.72	−1.0 (1.3)	.43	−1.9 (1.2)	.21
Fatigue^f^	−1.7 (1.5)	.24	−1.5 (1.7)	.38	−1.5 (1.2)	.18	−0.8 (0.9)	.37

Abbreviations: HE, health education; NA, not applicable; PST, problem-solving treatment.

^a^ Self-reported disability was assessed with the World Health Organization Disability Assessment Schedule 2.0. Higher scores indicate more disability (range, 1-100). Primary outcome was disability at 12 weeks.

^b^ Self-reported problem-solving impairment was assessed with the Problem Solving Inventory. Higher scores indicate greater problem-solving impairment (range, 32-192).

^c^ Objective problem-solving impairment was assessed with a composite score (mean *z* scores) of performance on a Stroop color word test, Trail Making Test B, Halstead Category Test–Russell revised, and Conners Continuous Performance Test. Lower scores indicate greater problem-solving impairment.

^d^ Pain was assessed with the Multidimensional Pain Inventory 3-item pain scale. Higher scores indicate greater pain (range, 0-18).

^e^ Pain disability was assessed with the Pain Disability Index. Higher scores indicate greater disability (range, 0-70).

^f^ Fatigue was assessed with the Fatigue Severity Scale. Higher scores indicate greater fatigue severity (range, 9-63).

Reduction in disability at 6 months was a secondary outcome. The PST group had a small reduction in disability at 6-month follow-up (PST: baseline mean [SE], 46.7 [1.9]; 6-month mean [SE], 44.1 [2.2]; Cohen *d* = 0.24, *P* = .07), whereas the HE group had a slight increase in disability (HE: baseline mean [SE], 45.1 [1.9]; 12-week mean [SE], 46.2 [2.1]; Cohen *d* = 0.15, *P* = .39). A moderate difference in reduction in disability was found between treatments at 6 months (Cohen *d* = 0.39, *P* = .06), suggesting that the PST group maintained reductions in disability, whereas the HE group went back to near baseline levels. Sensitivity analyses with imputed data supported a possible difference between the treatments at 6 months (Table 4).

### Problem-solving Impairment

The treatment × time interaction across time points was significant for self-reported problem-solving impairment (*F*_3,580_ = 4.12, *P* = .007), suggesting that changes in problem-solving impairment differed over time between the PST and HE groups. The PST group had a large reduction (Cohen *d* = 0.56, *P* < .001) (Table 2), whereas the HE group had a moderate reduction (Cohen *d* = 0.34, *P* < .001) in self-reported problem-solving impairment at 12 weeks. A moderate difference in reduction in self-reported problem-solving impairment was found between the treatments at 12 weeks (Cohen *d* = 0.33; *P* = .01) (Table 3), suggesting that PST resulted in greater reduction in self-reported problem-solving impairment compared with HE. This finding was supported by sensitivity analyses.

Problem-solving treatment led to a moderate reduction in self-reported problem-solving impairment at 6 months (Cohen *d* = 0.33, *P* = .001), whereas HE had no effect at 6 months (Cohen *d* = 0.07, *P* = .89). A moderate difference was found in reduction in self-reported problem-solving impairment between the treatments at 6 months (Cohen *d* = 0.42, *P* = .01), which suggested that PST maintained reductions in problem-solving impairment, whereas HE returned to near baseline levels. This suggestion was supported by sensitivity analyses.

The treatment × time interaction was not significant for objective problem-solving impairment (*F*_1,165_ = 0.75, *P* = .39), suggesting that changes in objective problem-solving impairment were similar between the PST and HE groups. The PST group had a small (Cohen *d* = 0.20, *P* = .07) and the HE group had a moderate (Cohen *d* = 0.31, *P* = .002) reduction in objective problem-solving impairment at 12 weeks (Table 2). Differences in reductions in objective problem-solving impairment between treatments at 12 weeks were similar (Cohen *d* = 0.09, *P* = .39) (Table 3), suggesting that PST did not reduce objective problem-solving impairment to a greater degree than HE. This outcome was supported by sensitivity analyses.

Mediational analysis showed that reduced self-reported problem-solving impairment mediated the relationship between PST and disability reduction (indirect effect, 1.55; 97.5% CI, 0.18-3.17; P_M_ = 2.62 for 12 weeks; indirect effect, 1.90; 97.5% CI, 0.26-3.84; P_M_ = 0.51 for 6 months). This finding suggests that reduced self-reported problem-solving impairment mediated disability reduction with PST.

### Pain, Pain Disability, and Fatigue

No differences were found in reduction of pain, pain disability, or fatigue between treatments at 12 weeks or 6 months (Table 3). Sensitivity analyses also did not reveal any consistent differences on these outcomes between treatments at 12 weeks or 6 months (Table 4).

## Discussion

The goal of this randomized clinical trial was to test whether PST would improve disability and problem-solving impairment in veterans with GWI compared with HE, an active control. We found no differences in the primary outcome, reductions in disability from baseline to 12 weeks, between PST and HE.

Although no meaningful differences were found between groups at 12 weeks, the overall mixed-model analysis for disability across all time points was significant. Results suggested that this was because PST sustained reductions in disability at 6 months, whereas disability levels in the HE groups returned to near-baseline levels (moderate effect). Caution is needed in interpreting this result, because the linear contrast did not reach statistical significance and data were missing at 6 months.

At 12 weeks and 6 months, PST reduced self-reported problem-solving impairment compared with HE (moderate effect). The meaningful reductions seen in problem-solving impairment in the PST group compared with the HE group may have enabled reductions in disability at follow-up for PST. We found reductions in problem-solving impairment–mediated reductions in disability for PST, suggesting the importance of targeting problem-solving impairment to improve long-term outcomes for GWI.

Of note, PST was acceptable to veterans with GWI. We found that 84.4% of veterans with GWI attended 100% of treatment sessions. This percentage is higher than in previous studies in which only 38% to 73% of veterans with symptoms consistent with GWI attended 40% to 60% of treatment sessions.^7,41,42^ We suspect the high acceptability is because the PST examined in this trial was tailored to veterans’ experiences with GWI. In addition, PST has been promulgated as an evidence-based practice in the VA, suggesting that PST could be disseminated to veterans with GWI through these trained providers.^43,44^

We unexpectedly found the acceptability of HE also to be high, likely because our HE was tailored for GWI.^25^ Furthermore, HE had a greater than anticipated immediate effect, which likely explained the lack of differences between groups at 12 weeks. However, the effects of HE on reductions in disability waned, suggesting the need for the future addition of behavioral support (eg, goal setting) to enhance its use as an active treatment, although further assessment is needed.^45^

We hypothesized, but did not find, that PST reduced objective problem-solving impairment, pain, and fatigue. Divergent self-report and objective problem-solving outcomes are consistent with findings from earlier clinical trials of PST^16,46^ and suggest the importance of using multidimensional assessments.^47^ The virtue of self-report is that it elicits the individual’s acknowledgment of relevant difficulties.^48^ In terms of pain and fatigue, our treatment was focused on reducing disability and was not designed to teach veterans symptom reduction skills. Treatments may need to specifically teach such skills to reduce pain and fatigue.

### Limitations

This study has some limitations. The generalizability of these results to other populations is not known. In addition, significant attrition was seen at 6 months, and 6-month results should be confirmed in future studies. Furthermore, although telephone delivery is generally efficacious, we did not assess the efficacy compared with face-to-face delivery for this population.

## Conclusions

The prespecified primary outcome of disability was not different between the PST and HE groups at the end of treatment in this randomized clinical trial. Secondary outcomes suggest that PST reduced problem-solving impairment and may have reduced disability at follow-up compared with HE, although this conclusion should be confirmed in future studies. Problem-solving treatment had high acceptability and is an evidence-based practice supported enterprise-wide in the VA.^44^ Together, the evidence that PST may reduce problem-solving impairment and disability at 6 months, has high acceptability, and is available in the VA, as well as the fact that there are few existing evidence-based treatments for GWI, suggests the potential for PST as a treatment for veterans with GWI.

## Notes

Supplement 1.
Trial Protocol and Statistical Analysis Plan

Supplement 2.
Data Sharing Statement
