# Supplementary material for: Effect of Problem-solving Treatment on Self-reported Disability Among Veterans With Gulf War Illness: A Randomized Clinical Trial
Source: JAMA Netw Open. 2022 Dec 6;5(12):e2245272. doi: 10.1001/jamanetworkopen.2022.45272 (PMC9856484; doi:10.1001/jamanetworkopen.2022.45272)
Supplement: Supplement 1. — Trial Protocol and Statistical Analysis Plan [file jamanetwopen-e2245272-s001.pdf]

Protocol Title: **Cognitive Rehabilitation for Gulf War Illness (GWI)**
PI: **Lisa McAndrew, PhD**
NCT02161133
War Related Illness and Injury Study Center
Version 8 – 08/02/2018

Background

Gulf War Illness (GWI) is a complex multi-dimensional illness which causes disability. Previous clinical trials (i.e. cognitive behavioral therapy and graded exercise) for GWI have sought to improve disability by increasing activity regardless of symptom presentation. These previous trials for GWI have shown limited efficacy and poor adherence. An innovative treatment approach is to target a specific component of GWI, namely problem-solving ability, known to be associated with disability.

Impairment in problem-solving ability affects Gulf War Veterans (GWV) with GWI and is prospectively related to greater risk of disability. This impairment is also related to poorer adherence to medical regimes, making it difficult for GWVs to manage other aspects of GWI. Problem-solving is considered one of the most complex of cognitive abilities and is related to complicated behaviors such as setting goals, sequencing and multi-tasking. Despite published reports documenting these deficits, there are no treatments that target the problem-solving deficits of GWI in order to reduce disability.

Problem-Solving Therapy

Problem-solving therapy (PST) is a targeted treatment to compensate for the problem-solving deficits of GWI and thereby reducing disability. PST, a top down approach, teaches patients skills to overcome problems like cognitive dysfunction or physical symptoms that impact problem-solving. Compensating for problem-solving deficits would reduce disability and provide information on the effect of treating one component of GWI on other symptoms of GWI.

PST is a treatment approach that teaches patients strategies to address real-life problems. It refers to the process by which people identify and implement effective means to cope with problems encountered in their lives. PST has been developed for individuals with problem-solving deficits. Traditionally, PST for cognitive rehabilitation has been 24 weeks long and is provided in group settings. PST for cognitive rehabilitation is split into two parts: problem-solving orientation phase and problem-solving skill phase and has been delivered over the phone to reduce attrition.
Aim 1: To determine the impact of telephone delivered Problem-Solving Therapy on disability in GWV with GWI. H1: Among Veterans with GWI, telephone delivered Problem-Solving Therapy will produce greater improvement in disability as compared to telephone delivered health education.

Aim 2: To determine the effect of Problem-Solving Therapy on problem-solving ability in GWV with GWI. H2a & H2b: Among Veterans with GWI, telephone delivered Problem-Solving Therapy will produce greater improvement in (H2a) self-reported problem-solving ability (measured with the Problem-Solving Inventory) and (H2b) objective problem-solving ability (measured with a neuropsychological battery) as compared to telephone delivered health education.

H2c & H2d: Improvement in (H2c) self-report problem-solving ability and (H2d) objective problem-solving ability will mediate the effect of telephone delivered Problem Solving Therapy on disability.

Exploratory Aim 3: To determine the effect of Problem-Solving Therapy on other symptoms of GWI. Problem-Solving Therapy may help GWV with GWI better compensate for other symptoms (e.g. avoid over exertion) which may improve other symptoms of GWI.
Exploratory H3a – H3b Among Veterans with GWI, telephone delivered Problem-Solving Therapy will produce greater improvement in (H3a) self-reported pain and (H3b) fatigue as compared to telephone delivered health education.

Study Population

We will screen up to 2,000 Veterans to obtain a final sample of up to 300 Veterans from the Gulf War who screen positive for GWI.

Inclusion:
(a) deployed to first Gulf War and meets Kansas definition for GWI (see definition in measures section);
(b) scores at least a half a standard deviation worse than the mean on the World Health Organization Disability Schedule (WHO-DAS II).

Exclusion:
(a) current suicidal/homicide intent or plan assessed by The Columbia Suicide Severity Rating Scale, schizophrenia or current psychotic symptoms
(b) self-reported diagnosis of a degenerative brain disorder or serious psychiatric or medical illness which may limit generalizability of the findings, limit safety or account for the symptoms of GWI.
Exclusionary medical illnesses include: Class 3 and 4 heart failure, cancer diagnosed within the past year and/or undergoing active treatment (chemotherapy or radiation therapy), chronic renal insufficiency, hospitalization due to myocardial infarct, stroke in the past year, a neurodegenerative disorder, or another medical or psychiatric disorder that may limit generalizability, limit participants safety or account for the symptoms of GWI at the discretion of the PI.
(c) a disability that would preclude telephone use.

Methods

The study is a collaborative randomized controlled trial with two arms: telephone delivered PST (treatment group) versus telephone delivered health education (control). Veterans with GWI will be recruited from all three sites: East Orange VA in New Jersey, Bedford VA in Massachusetts, and Canandaigua VA in New York. The East Orange VA will conduct all the telephone-delivered sessions. As needed, all sites, including East Orange VA, Bedford VA and Canandaigua VA, will assist with all parts of the study, e.g., mail follow-up questionnaires.

a. Recruitment and Screening

Potential participants (subjects) will be identified primarily via the Managerial Accounting Office (MCAO), VINCI, the Defense Manpower Data Center (DMDC) of the Department of Defense (DoD), and the VA Office of Public Health Gulf War Registry. We will ask the VINCI, the DoD, and the VA Office of Public Health Gulf War Registry to provide a list of Veterans who possibly meet our inclusion and exclusion criteria and also who reside in Region 1, and as needed nationally.

We will also use the following methods to identify and recruit potential participants: Referrals, standard mail, telephone, flyers, and advertisements. This includes via 1. planned conferences, satellite broadcasts, and other educational workshops for providers or Veterans; 2. VA facilities and affiliated ambulatory services including community-based outpatient clinics (CBOCs); 3. community locations (e.g., Home Depot); 3. Veteran related events such as job fairs; 4. newspapers/newsletters; 5. websites relevant to Gulf War Veterans (e.g., American Legions); and 6. VA social media; 7. WRIISC Clinical and Operations Database. We may also work with Veteran Service Organizations (VSO) to develop a recruitment plan. Veterans may be recruited from anywhere in the Nation.

Once potential participants are identified, introduction letters will be mailed to their addresses. Approximately after two weeks of mailing the introduction letter to Veterans, research staff will contact potential participants to inform them about the study, obtain verbal consent for and conduct initial screenings over the phone. For Veterans who contact us over the phone or in-person, (e.g., after reading a posted flyer), we will obtain verbal consent for and conduct intial screenings in person or over the phone.

If a Veteran is screened as eligible to participate in the study, a date and time will be scheduled for the Veteran to come into one of the three study sites (East Orange VA, Canandaigua VA or Bedford VA) to be consented into the study and complete the baseline questionnaires and neuropsychological assessments.

b. Sessions

Once consented and baseline assessments (described below) are completed, therapy sessions will be scheduled with a Study Provider from the East Orange VA. Study providers will be a licensed provider, e.g., psychologist, nurse, social worker, or a trainee e.g., working under a licensed provider, at the East Orange VA. A Study Provider from the East Orange VA will provide the sessions via phone with all participants from all VA sites (East Orange VA, Canandaigua VA, and Bedford VA). We will have a standardized assessment regimen that all Study Providers will adhere to. Therapy sessions will be scheduled weekly, and may be adjusted to accommodate the Veteran’s schedule and/or needs.

The telephone sessions with the Study Provider will generally be 1 hour. Most telephone sessions will be voice recorded and transcribed for quality assurance, i.e., fidelity to study protocols, length of sessions, engagement of participants. Coding of telephone sessions will occur by our off site collaborators once proper approvals are in place (i.e., DUA).

<u>Study Arms</u>

Veterans will be randomized either to the PST (treatment) or Health Education group (control).

If randomized to the PST (treatment):

A state of the art evidence-based 12 session PST intervention with a structured five step sequential approach to teaching problem-solving will be used. Our goal is to reduce disability (i.e., limitations in daily activities and participation). Study Providers will first work with participants to increase participation in the activities of their choosing. This could include increasing social activities, hobbies, physical activities, etc. Participants are then taught problem-solving skills and encouraged to problem-solve to overcome the barriers with increasing activity.

A 5-step problem-solving approach is employed whereby, 1) the problem is defined and goals are created, 2) possible solutions are generated, 3) these possible solutions are evaluated and one is selected, 4) the chosen solution is implemented in a step-by-step approach, and finally, 5) the outcome of the implemented solution is measured for success. Participants are introduced to the steps incrementally and each is reinforced by working with the Study Provider.

Participants will be given or mailed a PST workbook after the baseline assessment. For participants enrolled at the Bedford VA and Canandaigua VA, the Study Coordinator from the East Orange VA may mail the PST workbook. The content in the PST workbook will help guide participants through each session. The workbook also includes worksheets that are completed during each session and during the participant's own time. The workbooks will then be reviewed with the Study Provider during each session. At the Study Provider's or PI's discretion, the treatment or protocol may vary in order to meet a Veteran's needs (e.g., changing the order of the information given, having an additional session if feasible). During the sessions, participants will regularly be asked questions about their perception of the sessions, treatment, and if they have any new health concerns.

If randomized to the Health Education group (control):

Every week participants will receive up to an hour of didactic information and discussion about a variety of health concerns. Sessions will be highly structured and will emphasize the learning and analysis of key concepts in the presentations. This intervention will also be delivered over the telephone.

Participants will be given or mailed a Health Education workbook after the baseline assessment. For participants enrolled at the Bedford VA and Canandaigua VA, the Study Coordinator from the East Orange VA may mail the Health Education workbook. The Health Education workbook will be used in conjunction with the sessions. The content in the Health Education workbook includes didactic information on a variety of health topics pertinent to GWVs, i.e., pain, improving cognition, sleep, diet and physical activity. This information will also be reviewed with the Study Provider during each session. Similar to those randomized to the PST group, at the Study Provider's or PI's discretion, the Health Education protocol may vary in order to meet a Veteran's needs (e.g., changing the order of the information given, having an additional session if feasible).

Participants who are assigned to the health education control condition may be offered the 12-session PST program at the end of the study.

During the sessions, participants will regularly be asked questions on their perception of the sessions, treatment and if they have any new health concerns.

Reducing Attrition

To reduce attrition and the burden on the Veteran, all therapy sessions are done over the phone or video to phone if available. In addition, we plan to make phone calls to remind participants about their scheduled appointments (similar to the VA's automated appointment reminder system). PST has been successfully implemented over the telephone. Prior to starting the treatment, the participant and Study Provider may determine the time that is best for the Veteran (including nights and weekends) and may determine ground rules (for example, not having anyone else in the room, shutting off other telephones to remove distractions). The Study Provider and Veteran may also establish verbal signals to substitute for non-verbal cues, for example, "that's great" or "can I interrupt."

Participants will be allowed to begin or continue with their existing medical regimen throughout the course of the trial and make changes to their existing treatments. Adherence to medical protocols and participation in alternate treatments will be evaluated by a questionnaire at the assessments and this will be controlled for in analyses. The Study Provider may make additional recommendations for additional health care if needed or as appropriate.

We will make a concerted effort to contact participants for their appointments and assessments through phone calls and letters. We will not reach out to Veterans who do not wish to continue in the study.

Consent Procedures

Informed consent is an ongoing process. At the phone screening, Veterans will provide a verbal consent. At the baseline assessment, when the Veteran is considering enrolling in the study, information will be presented in-person to enable the Veteran to voluntarily decide whether or not to participate as a research participant. The Investigator, study coordinator or research assistant will review and explain the study's purpose, experimental procedures, alternatives, risks, benefits and duration of the study in terms that the potential participant can understand. The Investigator or study team member will be available to address any concerns and answer any questions the Veteran might have regarding the study and the informed consent. After all questions have been answered and concerns addressed, the Veteran may sign the Consent Form. A signed copy will be provided to the Veteran and the original copy will be placed in the Veteran's study folder and scanned into his/her CPRS record.

Participant Compensation

Participants may receive a total of $200 for their participation in the study as follows: $30 for completion of the baseline assessments; $70 for completion of the 12th session questionnaires and neuropsychological assessments. We will contact participants after the 4th session and also at 6 months post-treatment to complete written questionnaires – participants will receive $50 for completing each of these questionnaires. For compensation, participants will complete and sign a voucher, and a check will be mailed within 6-8 weeks. If the subject does not want to continue

in the study, s/he may come to an assessment site and complete a study exit visit and assessment; they will still receive $70.

Our budget includes funds to pay for participant travel as needed.

c. Assessment Methods

In this section we summarize all of the assessment instruments/procedures used to determine eligibility, assess neuropsychological functioning, and disability.

Table 2 outlines the study procedures that are described below: assessments, mailed questionnaires, qualitative interviews, and therapy sessions with the study provider.

		**Weeks**												**Months**
	**Baseline**	**1**	**2**	**3**	**4**	**5**	**6**	**7**	**8**	**9**	**10**	**11**	**12**	**6**
**Questionnaires**	X				X								X	X
**Neuropsychological Assessments**	X												X	
**Qualitative Phone Interview**	X												X	

Baseline and 12th Week Assessments

Baseline and 12th week assessments will be conducted in-person at any one of the study sites: East Orange VA, Canandaigua VA or Bedford VA. The baseline and 12th session assessments include completing written questionnaires and participating in neuropsychological assessments.

If the need arises (e.g., from fatigue) during the baseline or after the 12th session assessment, participants may have the option to complete the questionnaires at home and return them via mail in a prepaid envelope or complete the questionnaires via phone at a time and date that is convenient for him/her.

Participants may be provided the option of completing the written questionnaires and qualitative assessments only – and not completing the neuropsychological assessments. Questionnaire packets will still be mailed to them to complete and return to us in prepaid envelopes. Questionnaires may also be completed via phone with a study staff member at a time and date that is convenient for the participant. In addition, participants may be offered the opportunity to travel into one of the three study sites to complete the in-person baseline and 12th week assessments. We have budgeted this cost into the grant proposal.

<u>Special considerations for the Baseline Assessment only</u>
The baseline only will include full consenting into the study. Participants will also be asked to complete a mental health provider form as part of their baseline assessment. They will be encouraged to talk to their mental health provider and this provider form will be used to document it.

We will obtain the names of participants' primary care providers (PCP) from their medical records or form they will complete. We are asking for this information so we may inform PCPs

of their patients' participation in the study. This will enable a PCP to notify us of any health concerns s/he may have about his/her patient's participation in the study. At the PI's and/or Study Provider's discretion, we may also contact participants' health care providers at any time during the study if we have a concern about the Veteran's health and/or safety.

4th week and 6th month Assessment

We will mail or provide written questionnaires to all participants before or after the 4th session and also before or after 6 months post-treatment (see Table 1). All questionnaires will also be returned to the East Orange VA. Participants who wish, may also complete the questionnaires over the phone with a study staff member at a date/time that is convenient for the participant.

Qualitative Interviews

Participants may be asked to participate in 2 qualitative interviews. The qualitative interview will be conducted via phone by a study staff member from one of the sites at a date/time convenient for the participant after the baseline assessment and after the 12th session assessment. Participants will be asked about their perceptions of GWI and intervention methods. The qualitative interviews will be recorded using either a digital recording device or recorded directly onto a VA computer. Upon completion of all interviews, the recordings will be transferred to the secure shared network folder. The qualitative interviews will also allow us to modify the treatment in the future.

<u>Assessment Instruments</u>

a. Screening

The screening questionnaires will be used to determine inclusion and exclusion criteria. These questionnaires will be administered over the phone to determine a potential participant's eligibility, given exclusion and inclusion criteria, to enroll into the study. In addition to the listed questions we will ask potential participants about exclusionary medical and psychiatric conditions.

1. *<u>Case Definition of GWV' Illness (GWI) – Kansas Case Definition.</u>* The Institute of Medicine has recommended the use of the Kansas definition of Gulf War Illness(Steele, 2000). This definition was derived from a population-based survey of over 2,000 Veterans who served during the 1900-1991 Gulf War and whose symptoms began during or following deployment. The Kansas Case Definition identifies 6 symptom domains and inclusion requires that Veterans endorse moderately severe and/or multiple symptoms in at least 3 of those domains. The 6 symptom domains are: fatigue, pain, neurological/cognitive/mood, skin, gastrointestinal, and respiratory. To meet the case definition, Veterans must also indicate that each of those symptoms first became a problem during or after the Gulf War. Self-report of physician diagnosis of chronic conditions (e.g., cancer) that are not associated with Gulf War service, but that can produce diverse symptoms similar to those affecting GWVs (e.g., pain) are excluded under the Kansas Case Definition. This definition also excludes individuals with conditions that might interfere with respondents' ability to accurately report their symptoms (e.g., drug use). To develop the Kansas Case definition a narrow definition of conditions that might produce diverse symptoms was used. To improve

generalizability, we will only exclude participants with a clear disorder that could account for the GWI (e.g., multiple sclerosis).

2. *World Health Organization Disability Assessment Schedule (WHO-DAS 2.0):* The WHO-DAS 2.0 measures disability due to physical and mental health conditions (Ustun et al., 2010). The WHO-DAS 2.0 is a 36 item measure that focuses upon six life tasks:
   a. Understanding and communicating,
   b. Self-care
   c. Mobility (getting around)
   d. Interpersonal relationships (getting along with others)
   e. Work and household roles (life activities)
   f. Community and civic roles (participation)

   These six life tasks reflect two underlying constructs: Activity limitations and Participation deficits. The WHO-DAS 2.0 is based off of the International Classification of Functioning, Disability and Health (ICIDH-2), which categorizes disability into three categories: physiological, activities and participation. The WHO-DAS 2.0 has been translated into 15 languages and tested in 13 countries. The six domains have a factor loading of 0.82 to 0.98 and all load onto the general disability factor. Participants will be asked to complete this at all time points. We will use the WHO-DAS 2.0 screener which is a 12 item measure at screening. The WHO-DAS 2.0 screener explains 86% of the variance in the full measure and will reduce burden at screening. As the WHO-DAS is the primary outcome measure, we will use the full, and more valid, 36 item measure during the study.

3. *Columbia-Suicide Severity Rating Scale (C-SSRS):* The C-SSRS is the gold standard for suicide assessment. It is used by the VAs Center of Excellence for Suicide Prevention. The C-SSRS was developed by Columbia University and is used extensively across primary care, clinical practice, surveillance, research, and institutional settings. The C-SSRS in one study showed 100% sensitivity and specificity in identifying individuals who attempted suicide. Across three major randomized controlled trials, the C-SSRS demonstrated convergent, divergent, and predictive validity; sensitivity to change; sensitivity and specificity of the instrument; and internal consistency of the intensity subscale.(Posner et al., 2011) is part of a national and international public health initiative involving the assessment of suicidality, including general medical and psychiatric emergency departments, behavioral health organizations, medical homes, community mental health agencies, primary care, clergy, hospices, schools, college campuses, US Army, National Guard, VAs, Navy and Air Force settings, frontline responders, substance abuse treatment centers, prisons, juvenile justice systems, and judges to reduce unnecessary hospitalizations. At the screening and 12th session visit, this questionnaire will be administered by a member of the research staff.

b. Questionnaires

The questionnaires described by construct below will be administered at certain time points. Questionnaires may be administered in person at baseline and after the 12th session.

Questionnaires will be mailed or administered via phone after the 4thsessions, as well as 6 months post-treatment.

1. *Case Definition of GWV Illness (GWI) – Kansas Case Definition* (described above).

2. *The Fatigue Severity Scale (FSS):* The FSS is designed to differentiate fatigue from clinical depression, since both share some of the same symptoms (Takasaki & Treleaven, 2013). The FSS consists of a short questionnaire that requires the participant to rate his or her own level of fatigue. This measure has demonstrated good reliability, with a higher Cronbach's alpha indicated for the newer severity scale, as well as a correlation of 0.976 between the initial and current Fatigue Severity scales for a sample of 235 participants in seven groups. The FSS will be used to capture fatigue as a secondary outcome.

3. *Pain Disability Index (PDI) & Pain Inventory (MPI):* We will assess pain with 10 questions from two measures. We will first include the 7 item Pain Disability Index that assesses disability from pain. This measure has been found to be valid, have adequate test-retest reliability and be responsive to change in clinical trials (Soer et al., 2012; Tait et al., 1990). We will also use the 3 item West Haven Yale Pain Inventory that measures pain severity which has excellent test-retest reliability (0.74), internal consistency (.80), criterion validity, construct validity (Kerns et al., 1985). Both of these are commonly used together to characterize the suffering associated with pain.

4. *Patient Health Questionnaire (PHQ-15):* The PHQ is a self-administered questionnaire yielding algorithmic diagnoses of psychiatric illness. We will give the somatic symptoms (15 items) module. Patients are instructed to code each symptom based on whether it bothered them over the preceding 2 weeks: "not at all", "several days", "more than half the days"; or "nearly every day". The PHQ-15 was validated on a sample of 6,000 and found to be reliable, valid and responsive to change (Kroenke et al., 2002).

5. *MOS Sleep Scale and Insomnia Severity Index (ISI):* Medical Outcomes Study measure of sleep. This short version of the sleep scale is used to screen for sleep difficulties and social support. The internal reliability is .86 and there is adequate convergent and divergent validity (Hays et al., 1995). The ISI measures insomnia it has been shown to be reliable and valid (Gagnon et al., 2013). Together with the MOS Sleep Scale it provides an adequate measure of sleep disturbance in participants.

6. *World Health Organization Disability Assessment Schedule (WHO-DAS 2.0):* (described above)

7. *Veterans Rand 12 item Health Survey (VR-12):* The VF-12 is a multidimensional measure of disability that is commonly used among Veterans to understand impairment in activities. The VR-12 explains over 90% of the variance in the SF-36, a gold standard measure of disability. Test-retest correlations were excellent (.089 and .76) validity estimates were also excellent (Ware, Jr. et al., 1996).

8. *USER-P (activity frequency and satisfaction):* The USER-P captures satisfaction with activities and frequency of engaging activities, critical components of disability. The USER-P has been found to have adequate test-retest reliability. Further, in a comparison of three rehabilitation measures of disability, the USER-P was the measure most liked by participants (van der Zee et al., 2010)

9. *MOS Social Support:* The MOS Social Support measure captures if participants feel that they have access to available support to deal with and address problems in their life. This is another method to assess satisfaction with participation. The MOS Social Support is the best used measure of social support (2,500 citations to date) and has high convergent and divergent validity in addition to excellent reliability (Sherbourne & Stewart, 1991)

10. *Problem Solving Inventory (PSI)*: The PSI is a commonly used self-report measure of problem-solving. It is the measure that has been most used to assess changes in problem-solving during and after Problem-solving Treatment. The PSI measures self-assessed ability to deal with problems. The PSI has good internal consistency (alpha=.88) and excellent convergent and divergent validity (Sahin N et al., 1993).

11. *Behavioral Responses to Illness Questionnaire (BRIQ):* The BRIQ was developed to assess how individuals problem-solve medically unexplained symptoms. Internal reliability is above .80, test-re-test reliability is acceptable (.60-.87) and it is able to predict the onset of medically unexplained symptoms (Spence et al., 2005).

12. *Demographic Survey & Healthcare Access:* This is a brief questionnaire designed to elicit basic demographic information including: sex, age, marital status, self-identified racial and ethnicity group, educational level, income level, sources of current income, and current employment status, health insurance and health care utilization. The information obtained through this survey will be used to assess demographic differences between groups and identify potential control variables. We will also ask for self-report of medical conditions and medication.

13. *The Brief Traumatic Brain Injury Screen (BTBIS):* The VA/DOD BTBIS assesses TBI by asking if the participant has had an injury and if the injury resulted in a symptom. It will be used as a control variable in analyses.

14. *Patient Health Questionnaire (PHQ-8):* The PHQ is a self-administered questionnaire yielding algorithmic diagnoses of psychiatric illness. It was specifically developed and validated for use in primary care settings. We will administer the 8 item depression questionnaire. Patients are instructed to code each symptom based on whether it bothered them over the preceding 2 weeks: “not at all”, “several days”, “more than half the days”; or “nearly every day”. Depression is a common comorbid condition among Gulf War Veterans with Gulf War Illness.

15. *Post Traumatic Stress Disorder Checklist (PCL):* The 21 item National Center for PTSD Checklist of the VA will be used to assess PTSD. When compared to the gold standard for assessing PTSD, the Clinician Administered PTSD scale (CAPS), the PCL has been shown to be a valid and reliable measure of PTSD symptoms. PTSD is a common comorbid condition among Gulf War Veterans with Gulf War Illness.

16. *Alcohol Use Disorders Identification Test (AUDIT-C):* a 4-item alcohol screen used to identify patients with hazardous drinkers or have alcohol use disorders. The AUDIT is used in the VA to screen patients with alcohol use disorders.

17. *Illness Perception Questionnaire:* The illness perception questionnaire is a well validated and reliable measure of patient's illness cognitions. We have previously used this measure among Gulf War Veterans and found that it predicts adherence and satisfaction with medical care. We will use it to determine if the intervention changes cognitions. The illness perception questionnaire was developed to be modified for different populations and we have modified the questionnaire for use with this population. The Illness Perception Questionnaire has good internal reliability and long term test retest reliability. The IPQ also has good discriminant and predictive validity (Moss-Morris et al., 2002).

18. *Patient Global Impression of Change (PGIC):* This 2 item scale is widely used in clinical trials to gauge patient satisfaction. The scale asks the patient to rate status change since the beginning of treatment. It has been recommended as a core outcome among patients with medically unexplained symtpoms. This will be asked at the follow up time points only (Geisser et al., 2010).

19. *Working Alliance Inventory (WAI) – Self*: The working alliance is one of the most powerful predictors of change in therapy. It assess the participants perception of their relationship with their study provider. The working alliance has excellent reliability (.80) and convergent validity (Munder et al., 2010).

20. *Target Complaints Questionnaire (GOALS):* The measures ask participants what their current goals for treatment are and then if they meet them. This will enable us to understand the changes and if the treatment worked.

21. *Clients Satisfaction with Treatment:* This short validated measure (8 items) asks about clients' satisfaction with treatment. It is regularly used to assess satisfaction in clinical trials.

22. *MOS – Adherence Scale*: Assesses participants' perceptions of adherence to recommendations. This will assess if participants are following the treatment. The MOS Adherence Scale had adequate reliability and validity (Hays et al., 1995).

After the baseline visit and after the 12th session, participants may be asked about their perceptions of GWI and intervention methods. The qualitative interviews will be up to 45 minutes. This qualitative interview will also allow us to modify the treatment in the future.Qualitative interviews may be conducted on a subset of subjects enrolled over the duration of the project to achieve thematic saturation.

Qualitative assessments and interviews may be recorded, transcribed, and coded for further data analyses. Personal identifiers will not generally be transcribed from the voice recordings.

Additionally, we will review participants' medical records and collect information on previous and future medical treatment including physical and mental health treatment, diagnoses and medication.

c. Neuropsychological Measures

These measures capture Problem-solving Ability or Executive Function. These measures will be administered in-person at the baseline assessment visit and after the 12th session assessment visit. Problem-solving, an executive function refers to the ability of an individual experiencing an undesirable situation to move to a more desirable one when no response is immediately apparent or available. Problem-solving is considered the most complex of all cognitive functions as it requires the use and integration of other cognitive functions like attention, processing speed, effort, and impulse control as well as other executive functioning skills like mental flexibility and abstract thinking. Neuropsychological testing can be used to measure participants' functioning in the entire range of cognitive skills that problem-solving demands. We propose to use 5 neuropsychological instruments to measure functioning in the following cognitive areas: Attention, processing speed, impulse control, effort, mental flexibility and abstract thinking.

1. *Halstead Category Test, Russell Revised Version*: The Category Test is an objective test of problem-solving ability, specifically abstract thinking and mental flexibility. The test takes 30-40 minutes to administer. Our previous work has shown that the Category Test is able to differentiate Veterans with GWI from Gulf War Veterans without GWI. The Category Test has acceptable test-retest reliability and has been used as an outcome in cognitive rehabilitation treatments.

2. *The Conner's Continuous Performance Test-3 (CPT-3)*: The CPT-3 is a measure of ability to alternate attention,inhibit impulses and mental flexibility. The CPT-3 is a valid and reliable measure that and this version includes norms for adults with brain disorders as well as attentional disorders. It can and has been used in treatment efficacy trials.

3. *Stroop Color and Word Test:* The Stroop Test is a well-used measure of executive functioning and asks participants to read the color word (e.g., green) that is printed in black ink. Next, participants are asked to identify the color ink that "XXXX" is printed in (e.g., red). Finally, participants are asked to identify the color of the ink in which a word is printed (e.g., green) regardless of the color term being represented (e.g., blue). It is a measure of impulse control.

4. *Trails Making Test A and B (TMT):* Trails A and B asks participants to connect sequentially circles with different numbers and letters within them. It assesses processing speed a key component of problem-solving. .

5. *Rey-15, Memorization of 15-Items:* The Rey-15 is a short test of effort that instructs participants to look at and then remember 15 pieces of information. It is used as a control to ensure that participants are fully participating.

d. Study Provider Measures

At each assessment point, we will ask Study Providers to answer questionnaires about their views about the treatment for each Veteran they are providing treatment.

1. *Working Alliance Inventory (WAI) – Therapist*: The working alliance is one of the most powerful predictors of change in therapy. It has never been assessed among Gulf War Veterans and rarely in remote therapy. It assesses the relationship between the Study

Provider and participant. It is best assessed by asking both the provider and participant (Munder et al., 2010).

2. *SOFTA Questions:* The SOFTA asks Study Providers to answer questions about the behavioral indicators of the relationship between the Study Provider and participant.

3. *Target Complaints Questionnaire (GOALS):* The measures ask participants what their current goals for treatment are and then if they meet them. We have to capture what Veterans want to see changes in to understand if the treatment worked.

4. *Patient (Therapist) Global Impression of Change (PGIC):* We will adapt this measure for providers to measure their participant's impression of change to assess whether there is concordance between the provider's and participant's impressions.

5. *Concordance:* These measures assess whether a Study Provider's views of GWI are the same as the participant's views.

Subject Confidentiality/Data Safety and Monitoring

This is a collaborative clinical study that involves various components being administered from one of 3 sites: East Orange VA, Bedford VA, and Canandaigua VA. Recruitment will occur from all three sites. In addition, Veterans will consent and complete the baseline assessment and 12th session assessment in-person at one of the three sites. East Orange VA will provide the PST or health education via phone to all participants in the study. As needed, all sites, including East Orange VA, Bedford VA and Canandaigua VA, will assist with all parts of the study.

Data from all sites (East Orange VA, Canandaigua VA, and Bedford VA) will be kept on a VANJHCS server in a shared network folder created by IRM in East Orange, NJ. All data and study materials, including PHI from screening and recruitment, questionnaire data, neuropsychological data, and qualitative recordings and transcriptions will be stored and shared by all three sites via the shared network folder. This folder will have restricted access to study personnel approved on the protocol. Data will be collected in accordance with the protocol and under supervision of qualified personnel. Data may also be placed on the VA VINCI platform for data analysis.

There exists the possible risk of loss of confidentiality. We minimize these risks by assigning participants a unique study ID that is coded and which we will use on all electronic files resulting from their participation. The "link" or key that matches the ID code with participants' personal information will be kept in an electronic study folder on the server administered by IRM in East Orange in the IRM server room with access granted only to the PI and study staff across study sites.

In addition, all other paper documents, including signed consent forms, forms that ask for contact information, hard copies of data, and completed questionnaires, will be kept in a locked cabinet in room 11-198.

All recorded data from the therapy sessions and qualitative interviews will be stored on the network shared folder on the secure VA server with access limited to the research team. Before

being uploaded to the server, audio recorders will be stored in a locked cabinet with access to only study team members. The recordings may then be transcribed, with transcriptions stored on the same limited-access server. Transcriptions will generally contain no identifying information; basic information on gender and age range will be attached to each subject in the transcript, who will be identified with the assigned unique ID number (e.g., 10012).

The PI and study team members only will have access to the data and no confidential data will be shared with individuals outside of this team. Once a member of the study team leaves the study, s/he wil no longer have access to any of the data or study folders.

Data use agreements and proper approvals will be put into place with collaborators from outside institutions for data analyses.

Incident Reporting:
In the case that any digital recorders, electronic data, or hard copies of any research files and study data are compromised (e.g., lost, theft, unauthorized access, non-compliance with security controls), the PI will report the incident to the appropriate authorities/committees, including the VA NJHCS Information Security Officer and Privacy Officer within 1 hour of discovery, and will assist in the investigation and/or remediation of the incident.

Plan for destruction/return of data:
Study records will be destroyed in accordance with VHA RCS 10-1, and no less than 6 years past the date of study closure. All study records that are kept at the VA, including the links, will be destroyed in accordance with VHA RCS 10-1 and no less than 6 years past the date of study closure.

Data Analysis

a. Data

Data analyses will be conducted both on-site by study investigators and with proper approvals, including DUAs, at our affiliate institutes by current and future study investigators. All analyses will be primarily performed on an intention-to-treat basis. The statistical significance will be defined by a p value < 0.05, unless specified otherwise. Calculations of the means, standard deviations, proportions, histograms, etc., will be conducted to explore the distributions of the data, identify outliers, and calculate correlations between variables. We will also determine confounders that need to be controlled for in our analyses. We will particularly examine if we need to control for depression measured with the PHQ-8 or PTSD symptoms measured with the PCL based characteristics that exhibit differences at the baseline (pre-intervention).

Due to repeated measures, we will analyze the data using mixed model analysis, with participants nested within therapists modeled by random effects. To address H1, disability (WHODAS II scores) will be treated as the dependent variable, and treatment assignment [PST vs. Health education], time [pre- vs. post-intervention] and their interaction will be modeled as fixed effects. Differences in the improvement of disability between Problem-solving therapy and Health education will be evaluated by the treatment by time interaction. Linear contrasts will be constructed to evaluate the improvement in disability for each treatment separately. Variables such as age, gender, education and the confounders identified in the preliminary analysis will be controlled as covariates in the statistical analyses. Data transformations (e.g., square root, log,

etc.) will be used to normalize data where necessary. The same mixed model analysis strategy will be applied to address H2a, H2b, H3a and H3b. We will also explore, using this same mixed model strategy, the impact of PST therapy on each of the four individual neuropsychological tests scored as a composite and scored individually (Category Test, CPT 3, Stroop Test, and TMT). We will use the same data analysis strategy to examine all secondary outcomes. These will be reported as secondary outcomes and will primarily be used to generate hypotheses.

To address the H2c and H2d, we will establish a mediational model to explore whether the PST produces greater improvement in disability through its effects on problem-solving ability (measured by either the self-report (H2c) or the objective measurements (H2d)), following the recommendations of Baron and Kenny. The same mediational model will be used to explore whether participants views of treatment (e.g., relationship with therapist) also mediates the relationship between PST and disability. The statistical analysis will be performed using the mixed model analysis. Using these general methods we will also examine the impact of the treatment on other outcomes of interest, the relationship between variables and other potential moderators and mediators; we will also explore the relationship between variables of interest assessed concurrently.

b. Qualitative Data

Qualitative interviews will be transcribed and analyzed using standard qualitative research methods. The data to be analyzed are the participants' own words and narratives. Several members of the study team will perform an initial independent read through of interview notes and transcripts and generate a list of domains to create the initial coding scheme. Initial domains are expected to parallel the major themes or questions in the discussion and interview guides. Initial codes will be used to inform ongoing interviews and coding schemes. Once codes have been finalized, the coding scheme, interview notes and transcripts for interviews will be entered into an Excel or a qualitative software package. Study team members will apply the codes and labels to text.

Risks and Benefits

Risks

The risks of this study include:

1. Loss of confidentiality
2. Emotional distress. Some Veterans could find some of the questionnaires and the phone sessions upsetting.
3. As part of the study, Veterans will be encouraged to increase activities of their choosing. Examples of activities may include hobbies, household chores, social activities, or physical activity. Since the activities are personalized, the exact risks are not known. Participants are encouraged to talk with their primary care providers before engaging in activities. Study Providers will use clinical judgment to reduce risks as appropriate. Increasing activity (behavioral activation) is a standard component of cognitive and behavioral treatments. Veterans in the Health education component will be provided with didactic health information. All Veterans will be encouraged to talk with their PCPs prior to implementing any changes.

Benefits

This study will enable us to learn more about treatments that may help individuals with GWI. Some participants may experience improvement in the disability associated with GWI.

Reference List

Gagnon, C., Belanger, L., Ivers, H., & Morin, C. M. (2013). Validation of the Insomnia Severity Index in primary care. *J.Am.Board Fam.Med., 26,* 701-710.

Geisser, M. E., Clauw, D. J., Strand, V., Gendreau, R. M., Palmer, R., & Williams, D. A. (2010). Contributions of change in clinical status parameters to Patient Global Impression of Change (PGIC) scores among persons with fibromyalgia treated with milnacipran. *Pain, 149,* 373-378.

Hays, R. D., Sherbourne, C. D., & Mazel, R. (1995). *User's Manual for the Medical Outcomes Study (MOS) Core Measures of Health-Related Quality of Life*. Santa Monica, CA: RAND.

Kerns, R. D., Turk, D. C., & Rudy, T. E. (1985). The West Haven-Yale Multidimensional Pain Inventory (WHYMPI). *Pain, 23,* 345-356.

Kroenke, K., Spitzer, R. L., & Williams, J. B. (2002). The PHQ-15: validity of a new measure for evaluating the severity of somatic symptoms. *Psychosom.Med., 64,* 258-266.

Moss-Morris, R., Weinman, Petrie, & Horne (2002). The Revised Illness Perception QUestionnaire (IPQ-R). *Psychology and Health, 17*.

Munder, T., Wilmers, F., Leonhart, R., Linster, H. W., & Barth, J. (2010). Working Alliance Inventory-Short Revised (WAI-SR): psychometric properties in outpatients and inpatients. *Clin.Psychol.Psychother., 17,* 231-239.

Posner, K., Brown, G. K., Stanley, B., Brent, D. A., Yershova, K. V., Oquendo, M. A. et al. (2011). The Columbia-Suicide Severity Rating Scale: initial validity and internal consistency findings from three multisite studies with adolescents and adults. *Am.J.Psychiatry, 168,* 1266-1277.

Rath, J. F., Simon, D., Langenbahn, D. M., Sherr, R. L., & Diller, L. (2000). Measurement of problem-solving deficits in adults with acquired brain damage. *J.Head Trauma Rehabil., 15,* 724-733.

Sahin N, Sahin N H, & Heppner P (1993). Psychometric Properties of the Problem Solving Inventory in a Group of Turkish University Students. *Cognitibe Therapy and Research, 17*.

Sheehan, D. V., Lecrubier, Y., Sheehan, K. H., Amorim, P., Janavs, J., Weiller, E. et al. (1998). The Mini-International Neuropsychiatric Interview (M.I.N.I.): the development and validation of a structured diagnostic psychiatric interview for DSM-IV and ICD-10. *J.Clin.Psychiatry, 59 Suppl 20,* 22-33.

Sherbourne, C. D. & Stewart, A. L. (1991). The MOS social support survey. *Soc.Sci.Med., 32,* 705-714.

Soer, R., Reneman, M. F., Vroomen, P. C., Stegeman, P., & Coppes, M. H. (2012). Responsiveness and minimal clinically important change of the Pain Disability Index in patients with chronic back pain. *Spine (Phila Pa 1976.), 37,* 711-715.

Spence, M., Moss-Morris, R., & Chalder, T. (2005). The Behavioural Responses to Illness Questionnaire (BRIQ): a new predictive measure of medically unexplained symptoms following acute infection. *Psychol.Med., 35,* 583-593.

Steele, L. (2000). Prevalence and patterns of Gulf War illness in Kansas veterans: association of symptoms with characteristics of person, place, and time of military service. *Am.J.Epidemiol., 152,* 992-1002.

Tait, R. C., Chibnall, J. T., & Krause, S. (1990). The Pain Disability Index: psychometric properties. *Pain, 40,* 171-182.

Takasaki, H. & Treleaven, J. (2013). Construct validity and test-retest reliability of the Fatigue Severity Scale in people with chronic neck pain. *Arch.Phys.Med.Rehabil., 94,* 1328-1334.

Ustun, T. B., Chatterji, S., Kostanjsek, N., Rehm, J., Kennedy, C., Epping-Jordan, J. et al. (2010). Developing the World Health Organization Disability Assessment Schedule 2.0. *Bull.World Health Organ, 88,* 815-823.

van der Zee, C. H., Priesterbach, A. R., van der, D. L., Kap, A., Schepers, V. P., Visser-Meily, J. M. et al. (2010). Reproducibility of three self-report participation measures: The ICF Measure of Participation and Activities Screener, the Participation Scale, and the Utrecht Scale for Evaluation of Rehabilitation-Participation. *J.Rehabil.Med., 42,* 752-757.

Ware, J., Jr., Kosinski, M., & Keller, S. D. (1996). A 12-Item Short-Form Health Survey: construction of scales and preliminary tests of reliability and validity. *Med.Care, 34,* 220-233.

Summary List of Measures and Timeline

Domain	Study Instruments	# of Items	Screening	Baseline	4 week	12 week	24 week (6 month)
Screening	General Screening Questionnaire	27	X				
	Columbia Suicide Severity Rating Scale [C-SSRS]	26	X			X	
	Mini International Neuropsychiatric Interview [MINI]	23	X				
	World Health Organization Disability Assessment Schedule- 12 items [WHO-DAS]	12	X				
Estimated Completion Time for Screening Domain: 35-45 minutes							
Problem-Solving	Problem Solving Inventory [PSI]	32		X	X	X	X
	Behavioral Responses to Illness Questionnaire [BRIQ]	19		X	X	X	X
Estimated Completion Time for Problem-Solving Domain: 9 minutes							
Gulf War Illness	Fatigue Severity Scale [FSS]	9		X		X	X
	KANSAS Questionnaire [KANSAS]	29	X			X	
	Patient Health Questionnaire 15-items [PHQ-15]	15		X	X	X	X
	Pain Disability Inventory [PDI] & Pain Inventory [MPI]	10		X		X	X
	MOS Sleep Scale [MOS-Sleep] & Insomnia Severity Index [ISI]	19		X		X	
Estimated Completion Time for Gulf War Illness Domain: 16 minutes							
Disability	World Health Organization Disability Assessment Schedule- 36 items [WHO-DAS]	36		X	X	X	X
	Veterans RAND 12 Item Health Survey [VR-12]	12		X		X	
	USER-P (activity satisfaction and frequency) [USER-P]	21		X		X	X
	MOS Social Support [MOS-SS]	8		X		X	X
Estimated Completion Time for Disability Domain: 14 minutes							
Participant Characterization	Alcohol Use Disorders Identification Test Alcohol Consumption Questions [AUDIT-C]	4		X		X	
	Post Traumatic Stress Disorder Checklist [PCL-5]	20		X		X	X
	Patient Health Questionnaire 8 items [PHQ-8]	9		X		X	X
	Brief Traumatic Brain Injury [BTBIS]	4		X			
	Demographics [Demo]	30		X		X	X
	Healthcare Access [HCA]	17		X		X	X
Estimated Completion Time for Characterization Domain: 12 minutes							
Veterans Perceptions of Problem-Solving and Health Education	Working Alliance Inventory (Self) [WAI]	12			X	X	
	Target Complaints Questionnaire [GOALS]	6		X		X	
	MOS Adherence Scale [MOS-Adhere]	5		X		X	
	Patient Global Impression of Change [PGIC]	2				X	X
	Client Statisfaction Scale [Client Sat]	8				X	
Estimated Completion Time for Treatment Perceptions Domain: 8 minutes							
Veterans Perceptions of Gulf	Illness Perceptions Questionnaire [IPQ]	60		X		X	
Estimated Completion Time for Perceptions Domain: 10 minutes							
Total Estimated Completion for Questionnaires			35-45 mintue	70 minutes	20 mintues	70 minutes	35 minutes
Problem-Solving Neuropsychological Assessments	Halstead Category Test Short Form: Russell Revised Short Version [RCat]			X		X	
	Stroop Test			X		X	
	Trail Making Test Part A & B [TMT]			X		X	
	Continuous Performance Task [CPT-III]			X		X	
	Rey-15			X		X	
Estimated Completion Time for Neuropsychological Assessments: 50 minutes							
Qualitative Interviews				X (After Baseline is completed)		X (After Follow up is completed)	
Estimated Completion Time for a Qualitative Interview: 45 minutes							

Statistical Data Analysis Plan

Specific Aims:

Aim 1: To determine the impact of telephone delivered Problem-Solving Therapy on disability in GWV with GWI.
H1: Among Veterans with GWI, telephone delivered Problem-Solving Therapy will produce greater improvement in disability as compared to telephone delivered health education.

Aim 2: To determine the effect of Problem Solving Therapy on problem-solving ability in GWV with GWI.
H2a & H2b: Among Veterans with GWI, telephone delivered Problem-Solving Therapy will produce greater improvement in (H2a) self-reported problem-solving ability (measured with the Problem Solving Inventory) and (H2b) objective problem-solving ability (measured with a neuropsychological battery) as compared to telephone delivered health education.
H2c & H2d: Improvement in (H2c) self-report problem-solving ability and (H2d) objective problem solving ability will mediate the effect of telephone delivered Problem Solving Therapy on disability.

Exploratory Aim 3*: To determine the effect of Problem Solving Therapy on other symptoms of GWI.*
Problem-Solving Therapy may help GWV with GWI better compensate for other symptoms (e.g. avoid over exertion) which may improve other symptoms of GWI.
Exploratory H3a – H3b Among Veterans with GWI, telephone delivered Problem-Solving Therapy will produce greater improvement in (H3a) self-reported pain and (H3b) fatigue as compared to telephone delivered health education.

Data Analysis.

All statistical analyses will be primarily performed on an intention-to-treat basis. The statistical significance will be defined by a p-value < 0.05, unless specified otherwise. Calculations of the means, standard deviations, proportions, histograms, etc, will be conducted to explore the distributions of the data, identify outliers, and calculate correlations between variables. We will also determine confounders that need to be controlled for in our analysis. We will particularly examine if we need to control for depression measured with the PHQ-9 or PTSD symptoms measured with the PCL based characteristics that exhibit differences at the baseline (pre-intervention).

Due to the use of multiple therapists and repeated measures, we will analyze the data using mixed model analysis, with subjects nested within therapists modeled by random effects (e.g., SAS Proc Mixed). To address H1, disability (WHODAS II scores) will be treated as the dependent variable, and treatment assignment [Problem-Solving Therapy vs. Health Education], time [pre- vs. post-intervention] and their interaction will be modeled as fixed effects. Differences in the improvement of disability between Problem-Solving Therapy and Health Education will be evaluated by the treatment by time interaction. Linear contrasts will be constructed to evaluate the improvement in disability for each treatment separately. Variables such as age, gender, education and the confounders identified in the preliminary analysis will be controlled as covariates in the statistical analyses, where appropriate. Data transformations (e.g. square root, log, etc.) will be used to normalize data where necessary. The same mixed model analysis strategy will be applied to address H2a, H2b, H3a and H3b. We will also explore, using this same mixed model strategy, the impact of Problem-Solving Therapy on each of the four individual neuropsychological tests (Category Test, Wisconsin Card Sorting, PASAT and CPT).

To address the H2c and H2d, we will establish a mediational model to explore whether the Problem-Solving Therapy produces greater improvement in disability through its effects on problem-solving ability (measured by either the self-report (H2c) or the objective measurements (H2d)), following the recommendations of Baron and Kenny.[1] The statistical analysis will be performed using the mixed

model analysis. The conceptual framework for the analysis is outlined as follows: Three linear models will be constructed following the notation of Kraemer et al.[2]: let "O" denote the outcome variable (disability), "T" denote the independent variable (treatment) and "M" the mediators (problem-solving ability). The first model is the association model of T and M, i.e., $M=\gamma_0+\gamma_1 T+\varepsilon^*$ and the second model which is the meditational model: $O=\beta_0+\beta_1 T+\beta_2 M+\varepsilon$, where ε and ε^* are independent of T and M. The mediation effect will be evaluated by testing H_0: $\gamma_1\beta_2=0$, using the confidence interval (CI) approach.[3] If the 95% CI does not include 0, we will reject H_0 and establish the meditational model.

Dropouts and Missing Data. Dropout is common in longitudinal studies and can cause considerable bias if analyses are based only upon completed cases. Currently, there is no universally accepted statistical solution to the difficulties posed by missing data, especially when differential dropout in the treatment groups is present. In our study, we propose to use the likelihood-based statistical method (i.e., mixed model analysis) and this method generally provides valid statistical inference if the data are "missing at random" (MAR). When this assumption holds, we will use the standard approach such as the multiple imputation[4] to handle missing data. In cases where this assumption is tenuous, we will perform sensitivity analysis or explore the drop-out process and the missing data mechanism[5] such as the methods of selection models(e.g., [6]), or a latent variable approach (e.g., [7]) to assess the potential extent of the bias that may be caused by missing data.

4.4.3 Sample Size Estimation.

Previous studies of the effect of Problem-Solving Therapy on disability were used to make informed estimates of effect size. Alexopoulos found an improvement on the WHO-DAS 2.0 from 27.0 (SD=7.7) to 21.56 after 12 weeks, and the control intervention reduced the same measure from 26.2 (SD=7.0) to 23.70 after 12 weeks.[7] Assuming that our study will have a similar effect (e.g., the differences in the pre vs. post measurements between the Problem-Solving Therapy and Health Education control will be 2.94 with a common baseline variance of 7.7, equivalent to Cohen's d = 0.38), we estimated the sample size using the method of two sample t-test with alpha=0.05 (two-sided) and power=80%. Assuming the intra-subject correlation between the pre- vs. post intervention measurements to be 0.5, our study needs 109 subjects/group. After accounting for 15% attrition, we need 129 subjects per group (258 subjects in total). Applying the same power calculation to Aim 2 (H2a and H2b) our study has 80% power (alpha=0.05, two-sided) to test a Cohen's d of 0.38 in evaluating the effect of PST over HE on problem-solving ability.

1. Baron RM, Kenny DA. The moderator–mediator variable distinction in social psychological research: Conceptual, strategic, and statistical considerations. *Journal of personality and social psychology*. 1986;51(6):1173.
2. Chmura Kraemer H, Kiernan M, Essex M, Kupfer DJ. How and why criteria defining moderators and mediators differ between the Baron & Kenny and MacArthur approaches. *Health Psychology*. 2008;27(2S):S101.
3. MacKinnon DP, Fritz MS, Williams J, Lockwood CM. Distribution of the product confidence limits for the indirect effect: program PRODCLIN. *Behav Res Methods*. Aug 2007;39(3):384-9. doi:10.3758/bf03193007
4. Schafer JL. *Analysis of Incomplete Multivariate Data*. Monographs on Statistics and Applied Probability 72. Chapman & Hall/CRC; 1997.
5. Yang X, Shoptaw S. Assessing missing data assumptions in longitudinal studies: an example using a smoking cessation trial. *Drug and Alcohol Dependence*. 2005;77(3):213-225.
6. Molenberghs G, Verbeke G. A review on linear mixed models for longitudinal data, possibly subject to dropout. *Statistical Modelling*. 2001;1(4):235-269.
7. Albert PS, Follmann DA. A random effects transition model for longitudinal binary data with informative missingness. *Statistica Neerlandica*. 2003;57(1):100-111.
