# Supplementary material for: Effect of Problem-solving Treatment on Self-reported Disability Among Veterans With Gulf War Illness: A Randomized Clinical Trial
Source: JAMA Netw Open. 2022 Dec 6;5(12):e2245272. doi: 10.1001/jamanetworkopen.2022.45272 (PMC9856484; doi:10.1001/jamanetworkopen.2022.45272)
Supplement: Supplement 2. — Data Sharing Statement [file jamanetwopen-e2245272-s002.pdf]

Data Sharing Statement

McAndrew. Effect of Problem-Solving Treatment on Self-reported Disability Among Veterans With Gulf War Illness. *JAMA Netw Open*. Published December 06, 2022. doi:10.1001/jamanetworkopen.2022.45272

Data
Data available: No

Additional Information
Explanation for why data not available: Data will be made available upon request, when appropriate and consistent with informed consent and other participant permissions, and with permission from the Veterans Affairs.
